# Fungal Community Composition and Diversity Across Soil Depths Under Different Cover Crop Treatments

**DOI:** 10.3390/jof12020100

**Published:** 2026-01-31

**Authors:** Ephantus J. Muturi, Christopher A. Dunlap, Jose L. Ramirez, William L. Perry, Nicholas Heller, Robert L. Rhykerd

**Affiliations:** 1 U.S. Department of Agriculture, Agricultural Research Service, National Center for Agricultural Utilization Research, Crop Bioprotection Research Unit, 1815 N. University St., Peoria, IL 61604, USA; christopher.dunlap@usda.gov (C.A.D.); jose.ramirez2@usda.gov (J.L.R.); 2 School of Biological Sciences, Illinois State University, Normal, IL 61670, USA; wlperry@ilstu.edu; 3 Department of Agriculture, Illinois State University, Normal, IL 61670, USA; njhelle@ilstu.edu (N.H.); rrhyker@ilstu.edu (R.L.R.)

**Keywords:** fungal community, soil depth, cover crops, trophic mode, soil properties, mycobiome

## Abstract

Fungi are a critical component of microbial biomass in agricultural soils, but their distribution across soil depths under different cover crops remains poorly understood. We used high-throughput sequencing of fungal ITS1 amplicons to characterize fungal communities across four soils depths (0–2, 2–4, 4–10, and 10–20 cm) in experimental field plots under four cover crop treatments: winter fallow reference (REF), cereal rye (CRYE), wild pennycress (WPEN), and a mixture of pea, crimson clover, radish, and oat (PCRO). There was no significant interaction between soil depth and cover crop treatment on both alpha diversity and beta diversity. CRYE and PCRO cover crops had low abundance of *Fusarium*, a genus including many important plant pathogens, and different fungal community composition relative to REF. Fungal diversity was significantly higher at 4–10 cm compared to 0–2 cm depth, but fungal richness was not affected by soil depth. Fungal community composition differed significantly between 0–4 and 10–20 cm soil depths. The relative abundance of *Mortierella* and unclassified Basidiomycota increased with increasing soil depth while that of *Calvatia*, *Cryptococcus*, *Fusarium*, and *Idriella* decreased with increasing soil depth. Most fungal taxa were assigned to more than one guild, but the few taxa that were classified as strict saprophytes decreased with increasing soil depth while those classified as strict symbionts increased with increasing soil depth. These differences were associated with low pH and high content of OM, K, S, P, and Zn in the topsoil layer compared to the deeper soil layer. The findings may inform the development of targeted soil management practices to promote beneficial fungi, but additional studies covering multiple study sites and sampling dates are needed for clarity.

## 1. Introduction

Soil fungi are crucial to the overall health and functioning of agricultural systems. Although fungi are mostly known for causing devastating plant diseases, they are also key contributors of litter decomposition and nutrient cycling and can promote plant health by suppressing plant pathogens and insect pests, and improving plant access to nutrients [1,2,3,4]. Soil fungi also play an important role in microbial food webs by linking carbon and nutrient flow between primary producers and high-order consumers [5]. Therefore, an in-depth understanding of the factors that influence the diversity and composition of soil fungal communities is needed to facilitate the development of effective strategies to enhance soil health and sustainability of agricultural systems.

Numerous studies have evaluated how different agricultural practices (e.g., cover cropping, fertilization, and no till) influence the diversity and structure of soil fungal communities. Most of these studies focus on composite samples of the top 0–25 cm soil depth because it contains most of the plant roots and exhibits higher microbial diversity, organic matter, and nutrient concentrations [6,7,8]. However, a variety of factors that are known to influence soil fungal diversity and composition including litter quality and quantity, root-derived carbon, soil pH, nutrients, texture, and water content vary widely across soil depths [9,10,11], causing shifts in fungal community composition and a decrease in fungal abundance and diversity with increasing soil depth [12,13,14]. Recent studies from the same experimental plots revealed that these effects are especially prevalent and detectable at shallow soil depths of 0–4 cm [15]. These findings suggest that some important effects of soil depth on fungal communities are likely missed when soil samples from the top layer (0–25 cm) are combined and analyzed as a homogeneous unit.

Cover cropping is one of the soil management practices that is increasingly used to improve soil health while boosting crop yield. In addition to preventing soil erosion between cropping seasons, cover crops provide a multitude of other essential services. These include soil enrichment with organic matter and nutrients, accelerated sequestration of soil carbon, suppression of weeds, insect pests, and soil-borne pathogens, improvement in soil porosity and water holding capacity, and enhancement of soil microbial activity, diversity, and biomass [16,17,18,19,20,21,22,23,24,25,26]. Plants in Poaceae (grass), Brassicaceae (mustard), and Fabaceae (legume) families dominate the list of the most-used cover crops, but other families such as Boraginaceae (the borage) and Asteraceae (daisy) are also utilized. Plants from these families are known to differentially affect the physical, chemical, and biological properties of the soil [27,28] including soil microbial diversity, composition, and abundance [6,8,29]. Numerous studies have evaluated the impact of cover crops on soil fungal community structure and diversity. These studies have shown that some cover crop species can alter soil fungal structure and reduce, increase, or have no effect on fungal diversity and richness [6,9,10,15,29]. However, our understanding of how different cover crop species influence soil microbial communities at different soil depths remains incomplete, particularly for fungal communities.

We used high-throughput DNA sequencing to explore how soil depth affects the diversity and composition of fungal communities in field plots planted with four cover crop treatments (winter fallow reference plot, cereal rye (*Secale cereale*), wild pennycress (*Thlaspi arvense*), and a mix of pea, crimson clover, radish, and oat (*Pisum sativa*, *Trifolium* sp., *Raphanus sativus*, *Avena sativa*). The study expands our earlier analysis from 0–4 cm depth [15] to a deeper profile of 0–20 cm. We tested the hypotheses that (1) fungal community composition would differ across soil depths and fungal diversity and richness would decrease with increasing soil depth; (2) fungal guilds will be stratified across soil depths; and (3) different cover crops will have variable effects on fungal communities at different soil depths.

## 2. Materials and Methods

### 2.1. Collection Sites

Soil samples were collected at Illinois State University research farm at Lexington, IL, USA (40.674641, −88.783492) from Catlin, Flanagan, and Drummer soil series. All three soil types were formed in loess material and consist of silt loam and silty clay loam soil textures [30]. The Catlin soils series (Fine-silty, mixed, superactive, mesic Oxyaquic Argiudolls) is a very deep, moderately well-drained soil. The Flanagan soil series (Fine, smectitic, mesic Aquic Argiudolls) and Drummer soil series (Fine-silty, mixed, superactive, mesic Typic Endoaquolls) are composed of very deep, somewhat poorly drained soils. The study did not require any permits and complied with all relevant regulations. A detailed description of experimental plots is reported elsewhere [15]. In brief, experimental plots measuring 4.6 m × 12.2 m were established on 17 October 2020, in a randomized complete block design with four blocks. Plots within each block were randomly assigned and seeded with one of four cover crop treatments: a winter fallow reference plot (REF), cereal rye (CRYE), wild pennycress (WPEN), and a mixture of pea, crimson clover, radish, and oat (PCRO). Each plot has been seeded with the same cover crop every year since 2020 and no fungicides have been used on any crop in these plots. On 10 June 2024, soil samples were collected from the subplots using a handheld soil probe. The soil core was subsampled at four soil depths (0–2, 2–4, 4–10, and 10–20 cm) and four cores were collected for each depth per plot and combined into a single composite sample. These depths were chosen based on our previous findings that fungal community composition differed significantly between 0–2 and 2–4 cm soil depths [15]. The soil samples were freeze-dried, and 20 g of each sample was homogenized in a blender (Waring Inc., Stamford, CT, USA) until finely ground and preserved at −80 °C until further processing.

### 2.2. Soil DNA Extraction and Sequencing

The methods used in DNA extraction and sequencing are detailed elsewhere [15]. In brief, soil samples were lyophilized; then, 20 g of each sample was homogenized in a blender for 10 s. DNA was extracted from 200 mg of homogenized soil samples using DNeasy PowerLyzer PowerSoil Kit (Qiagen Inc., Hilden, Germany) following the manufacturer’s protocol. Molecular-grade water was used as a negative control. DNA concentration and quality were determined using NanoDrop (Thermo Fisher Scientific, Waltham, MA, USA). The ITS1 region of the ribosomal RNA gene was PCR-amplified using the primer set ITS1F and ITS2 [31] that was added at the end of Illumina overhang adapter sequences. Each PCR reaction had a total volume of 30 µL consisting of 15 µL of Amplitaq Gold 360 master mix (ThermoFischer Scientific Inc.), 1.5 µL of each primer, and 12 µL of template DNA. The PCR program consisted of an initial denaturation at 95 °C for 10 min, 35 cycles of 95 °C for 30 s, annealing at 58 °C for 30 s, and extension at 72 °C for 60 s. The size and quality of PCR amplicons were analyzed on a 2% agarose gel using gel electrophoresis and purified using Ampure XP beads (Beckman Coulter, Brea, CA, USA). A second PCR reaction to attach dual indices and Illumina sequencing adapters was conducted as previously described [32]. Cleaned DNA libraries were normalized using a SequalPrep^TM^ normalization plate (Thermo Fisher Inc., Waltham, MA, USA), pooled, and quantified with a Kapa library quantification kit (Kapa Biosystems Willington, MA, USA). Sequencing was conducted on an Illumina MiSeq system using 2 × 300 bp paired-end sequencing. The raw data is available at GenBank under BioProject PRJNA1293797.

### 2.3. Measurement of Soil Chemical and Physical Properties

Quantification of the physical and chemical properties of the soil samples was conducted at the Element Agriculture laboratory in Centralia, IL, using the Recommended Chemical Soil Test Procedures for the North Central Region [33]. These procedures are described in detail elsewhere [15]. The soil properties that were analyzed included soil organic matter (OM), cation exchange capacity (CEC), pH, phosphorus (P), potassium (K), sodium (Na), calcium (Ca), magnesium (Mg), sulfur (S), zinc (Zn), manganese (Mn), copper (Cu) boron (B), and iron (Fe).

### 2.4. Data Analysis

Raw DNA amplicon sequences were processed using QIIME2 (version 2023.2) bioinformatics pipeline [34]. The divisive amplicon denoising algorithm (DADA2) plugin in QIIME2 was used to filter and trim the reads, dereplicate the filtered reads, learn the error rates, remove chimeric sequences, merge forward and reverse reads, and create the sequence table [35]. Default options were used to implement the denoise-paired command except for the following parameters that were applied to remove the primers and truncate read length: trim-left-f 22, trim-left-r 20, trunc-len-f 224, trunc-len-r 224. After this filtering step, 72–91% of raw reads were retained for downstream analysis. QIIME2 feature classifier and UNITE database v7.2 were used to assign taxonomy for each amplicon sequence variant [36]. No obvious contaminants were detected in the samples based on assessment of the negative controls.

The Phyloseq package in R version 4.4.1 [37] and PAST 4.03 statistical software [38] were used for statistical analysis. For beta diversity analysis, raw data was transformed to relative abundance and non-metric multidimensional scaling (NMDS) with Bray–Curtis dissimilarity matrices was performed using metaMDS function in vegan R package version 2.6-6 (hereafter vegan) to visualize the relative effects of soil depth and cover crop treatments on fungal communities [39]. Envfit function with Monte Carlo permutation test (999 permutations) was used to determine the association between changes in fungal community composition and the measured soil properties. Soil data was first checked for multicollinearity and variables with a variance inflation factor greater than 10 were removed. These included CEC, Na, Ca, and Fe. The variables that were not significantly different between soil depths (i.e., Mg, Mn, and Cu) were also excluded from the model. The effects of cover crop treatment and soil depth on fungal communities was evaluated using permutational multivariate analysis of variance (PERMANOVA) performed with “adonis2” function in vegan [39] and corrected for multiple comparisons using Bonferroni test. Block was included as a random effect in the PERMANOVA model. When stratified by block, PERMANOVA cannot test both main effects and their interaction due to loss of permutation freedom. Therefore, two separate models were fit, one for the main effects, and one for interaction. Dispersion test was conducted using the “betadisper” function in vegan. SIMPER analysis using PAST statistical software was used to evaluate the fungal taxa that contributed most to the observed differences between treatments. The UpSetR package version 1.4.0 was used to generate the upset plot to visualize patterns of unique and shared fungal ASVs.

To assess the impact of soil depth and cover crop treatments on alpha diversity, the sequences were rarefied to the minimum library size (17,594 reads per sample) to standardize the sampling effort. Vegan package was used to compute the observed ASVs (richness) and Shannon diversity index for each sample. The resulting data was checked for normality using Shapiro–Wilk test, and Shannon diversity index which failed this test was log-transformed prior to analysis. Generalized linear (GL) models and linear mixed-effects (LME) models were used to evaluate the effect of cover crop treatment and soil depth on fungal richness (observed ASVs) and Shannon diversity. Cover crop treatment, soil depth, and their interactions were included as fixed effects with block as a random effect in linear mixed-effects models. Tukey’s HSD test was used to evaluate means between treatments. For observed ASVs data, the LME model with AIC value of 667.7 was not a significantly better fit for the data compared to the GL model with AIC value of 666.5 (Chisq = 0.7631, *p* = 0.3823). Therefore, the GL model was used for data interpretation. No LME model was found to fit the Shannon diversity data and, thus, Bayesian generalized non-linear multivariate multilevel was conducted using the R package “brms” version 2.22.0. Putative functional characteristics of fungal ASVs (i.e., Pathotrophs, Saprotrophs, Symbiotrophs, and multiple trophic modes) was assigned using FUNGuildR version 0.3.0 [40]. Only guilds assigned as “highly probable” and “probable” were considered.

Multivariate analysis of variance (MANOVA) was used to determine the effect of soil depth on the 10 physical and chemical properties of the soil that met the assumption of multicollinearity. Organic matter values were arcsine transformed and the remaining 9 variables were log(x + 1) transformed to meet the assumptions of multivariate normality. Tukey’s HSD test was used to determine which pairs of means were significantly different. Although CEC, Na, Ca, and Fe were excluded in MANOVA and NMDS models, they were analyzed separately and their means were reported along with those of variables that were included in the two models.

## 3. Results

### 3.1. Impact on Alpha Diversity

**Figure 1:**
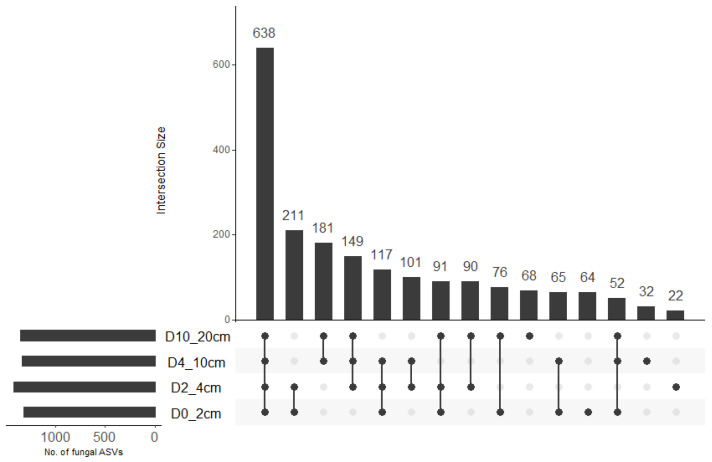
Upset plot showing the number of fungal ASVs that were unique to specific soil depth or shared between soil depths. The connected dots indicate the common different ASVs across intersecting soil depths.

A total of 2,092,084 sequences were obtained across 64 samples. The total number of sequences per sample ranged from 17,594 to 46,523 with a mean (±SE) of 32,688.8 ± 750.5. A total of 1957 amplicon sequence variants (ASVs) were identified across all samples. There was no significant effect of soil depth (F = 0.519, df = 3, 48, *p* = 0.671), cover crop treatment (F = 0.857, df = 3, 48, *p* = 0.470), or their interaction (F = 1.975, df = 9, 48, *p* = 0.063; R^2^ = 0.31) on fungal ASV richness. Fungal ASV diversity (Shannon diversity) was significantly influenced by soil depth (*x*^2^ = 15.313, df = 3, *p* = 0.002) but not cover crop treatment (*x*^2^ = 6.142, df = 3, *p* = 0.105). Fungal diversity was significantly higher at 4–10 cm compared to 0–2 cm depth (Table 1). Upset plot indicated that 64, 22, 32, and 68 fungal ASVs were unique to 0–2 cm, 2–4 cm, 4–10 cm, and 10–20 cm soil depths, respectively (Figure 1). These accounted for 3.3%, 1.1%, 1.6%, and 3.5% of the total ASV pool. The largest intersection size represented 638 fungal ASVs that were shared across all soil depths while the second-, third- and fourth-largest intersection sizes represented fungal ASVs that were shared between 0–2 cm and 2–4 cm depths (211), 4–10 and 10–20 cm depths (181), and 2–4 cm, 4–10 cm, and 10–20 cm depths (149), respectively. This accounted for 32.6%, 10.8%, 9.2%, and 7.6% of the total ASV pool.

### 3.2. Phylum and Genus Composition

**Figure 2:**
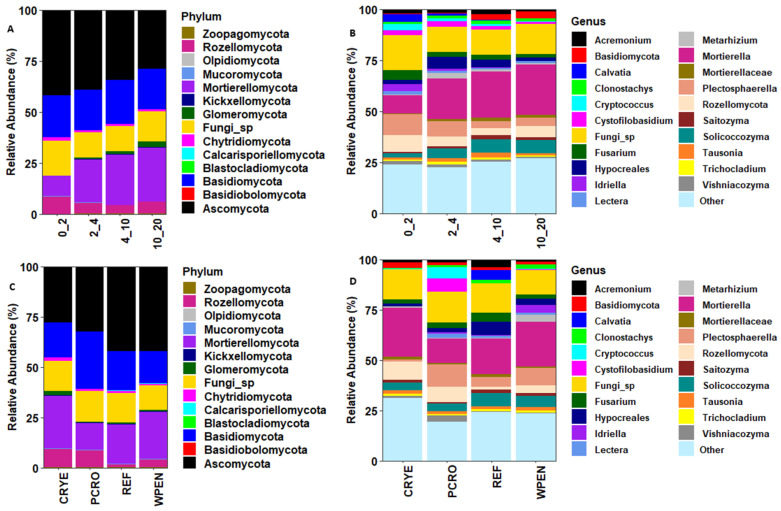
Relative abundance of soil fungal communities at phylum and genus levels in relation to soil depth (**A**,**B**) and cover crop treatments (**C**,**D**). CRYE (cereal rye), PCRO (a mixture of pea, crimson clover, radish, and oat), REF (winter fallow reference), and WPEN (wild pennycress).

The seven most abundant phyla across all cover crop and soil depth treatments accounted for 99.3% of the total sequences. These included Ascomycota (38.5%), Basidiomycota (20.6%), Mortierellomycota (18.5%), unclassified Fungi (14.0%), Rozellomycota (5.9%), Chytridiomycota (1.2%), and Glomeromycota (0.7%). Other fungal phyla including Mucoromycota, Kickxellomycota, Zoopagomycota, Blastocladiomycota, Basidiobolomycota, Olpidiomycota, and Calcarisporiellomycota accounted for 0.6% of the total sequences (Figure 2). The relative abundance of phylum Ascomycota decreased with increasing soil depth (42.0%, 39.1%, 34.4%, and 28.9%) while the relative abundance of Mortierellomycota (10.0%, 21.2%, 24.4%, and 26.3%) and Glomeromycota (0.2%, 0.5%, 1.3%, and 2.5%) increased with increasing soil depth. The relative abundances of Basidiomycota (20.5%, 19.9%, 21.3%, and 19.8%) and Chytridiomycota (1.4%, 1.1%, 1.1%, and 1.0%) were similar across soil depths. The relative abundance of Rozellomycota was higher at 0–2 cm soil depth compared to other soil depths (8.5% vs. 5.1%, 4.1%, and 5.8%).

At the genus level, the 10 most abundant taxa across all treatments were *Mortierella* (19.0%), *Fungi* sp. (14.2%), *Plectosphaerella* (6.4%), unclassified *Rozellomycota* (5.5%), *Solicoccozyma* (4.9%), unclassified Hypocreales (3.4%), *Fusarium* (2.8%), *Acremonium* (2.0%), *Cystofilobasidium* (2.0%), and unclassified Basidiomycota (1.7%, Figure 2). The relative abundance of genus *Mortierella*, *Solicoccozyma*, and unclassified Basidiomycota increased with increasing soil depth while the relative abundance of *Fusarium* decreased with increasing soil depth. The relative abundance of *Plectosphaerella* was higher at 0–2 cm (10.4%) and 2–4 cm (7.6%) soil depths compared to 4–10 cm (3.4%) and 10–20 cm (4.2%) soil depths. The relative abundance of unclassified Rozellomycota was higher at 0–2 cm soil depth (8.2%) compared to other soil depths (4.7%, 3.5%, 5.5%). The relative abundance of unclassified Hypocreales was higher at 2–4 cm and 4–10 cm soil depths relative to 0–2 cm and 10–20 cm soil depths. The relative abundance of *Acremonium* (2.2%, 1.9%, 2.5%, and 1.2%) and *Cystofilobasidium* (2.4%, 2.7%, 1.6%, and 0.9%) was lower in 10–20 cm soil depth compared to other soil depths.

For the cover crop treatment, the relative abundance of Ascomycota was higher in REF (42.0%) and WPEN (42.2%) compared to CRYE (28.0%) and PCRO (32.2%). The relative abundance of Mortierellomycota was highest in CRYE (25.8%) and WPEN (23.4%), intermediate in REF (19.3%), and lowest in PCRO (13.3%). The relative abundance of Basidiomycota was highest in PCRO (28.8%), intermediate in REF (19.7%) and CRYE (17.2%), and lowest in WPEN (16.0%). The relative abundance of Rozellomycota was highest in CRYE (9.3%) and PCRO (8.4%), intermediate in WPEN (4.0%), and lowest in REF (1.7%). The relative abundance of Chytridiomycota was highest in CRYE (1.6%) and lowest in PCRO (0.8%), while the relative abundance of Glomeromycota was higher in CRYE (2.4%) compared to the other cover crop treatments.

At the genus level, the relative abundance of *Mortierella* was highest in CRYE (24.2%) and WPEN (22.1%), intermediate in REF (17.6%), and lowest in PCRO (12.2%). The relative abundance of *Plectosphaerella* was highest in PCRO (11.0%) and WPEN (8.5%), intermediate in REF (4.7%), and lowest in CRYE (1.3%). The relative abundance of Rozellomycota was highest in CRYE (9.0%) and PCRO (7.7%), intermediate in WPEN (3.8%), and lowest in REF (1.5%). The relative abundance of *Solicoccozyma* was higher in REF (6.7%) and WPEN (5.6%) than in CRYE (3.8%) and PCRO (3.6%). The relative abundance of Hypocreales was highest in REF (6.8%), intermediate in WPEN (3.2%) and PCRO (2.1%), and lowest in CRYE (1.4%). The relative abundance of *Fusarium* was higher in REF (4.4%) than in CRYE (2.0%), PCRO (2.6%), or WPEN (2.3%).

### 3.3. Impact on Beta Diversity

**Figure 3:**
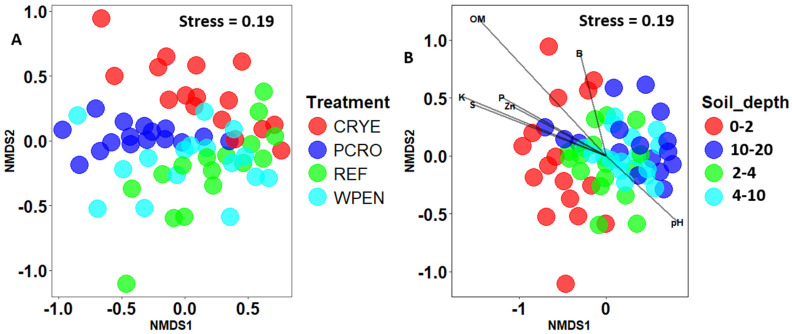
Non-metric multidimensional scaling (NMDS) of Bray–Curtis distances between fungal communities from (**A**) four cover crop treatments and (**B**) four soil depths. CRYE (cereal rye), PCRO (a mixture of pea, crimson clover, radish, and oat), REF (winter fallow reference), and WPEN (wild pennycress). Envfit was not conducted for cover crop factor since only one variable was significantly associated with cover crop treatment.

Non-metric multidimensional scaling analysis of Bray–Curtis distances showed some separation between cover crop treatments and soil depths (Figure 3). PERMANOVA analysis revealed significant effects of cover crop treatment (F = 5.315, df = 3, 57, *p* = 0.001) and soil depth (F = 4.693, df = 3, 57, *p* = 0.001), but not their interaction (F = 0.792, df = 9, 48, *p* = 0.793) on beta diversity. Pairwise contrasts for cover crop treatments showed significant differences between REF vs. CRYE and REF vs. PCRO cover crop treatments, while pairwise contrasts for soil depth showed that fungal communities at 10–20 cm soil depth were significantly different from those of 0–2 cm and 2–4 cm soil depth. Dispersion test was significant for both cover crop treatment (F = 2.99, df = 3, 60, *p* = 0.04) and soil depth (F = 4.46, df = 3, 60, *p* = 0.01) but not their interaction (F = 1.09, df = 15, 48, *p* = 0.387).

SIMPER analysis was used to identify the fungal taxa that contributed to these differences (Table 2). Ten genera accounted for 58.8% of the observed differences in fungal communities between soil depths. These included *Mortierella* accounting for most of variation (19.2%), unclassified fungi (9.2%), unclassified Hypocreales (5.6%), *Cystofilobasidium* (4.5%), *Cryptococcus* (3.8%), unclassified Basidiomycota (3.8%), *Fusarium* (3.6%), *Calvatia* (3.4%), *Idriella* (3.1%), and *Metarhizium* (2.7%). *Mortierella* and unclassified Basidiomycota increased with increasing soil depth while *Calvatia*, *Cryptococcus*, *Fusarium*, and *Idriella* decreased with increasing soil depth. The relative abundance of unclassified Hypocreales and *Cystofilobasidium* was highest in the 2–4 cm soil depth, intermediate in 0–2 cm depth, and lowest in 10–20 cm depth. The relative abundance of *Metarhizium* was highest in 2–4 cm depth and lowest in 0–2 cm depth.

SIMPER analysis for cover crop treatments revealed the main genera accounting for the observed dissimilarity to be *Mortierella* (18.3%), unclassified fungi (9.1%), Hypocreales (5.6%), *Cystofilobasidium* (5.3%), *Cryptococcus* (4.2%), *Fusarium* (3.6%), unclassified Basidiomycota (3.5%), *Calvatia* (3.5%), *Idriella* (3.1%), *Metarhizium* (2.7%), *Acremonium* (2.5%), and *Lectera* (2.2%), which collectively accounted for 63.6% of the variance (Table 2). The relative abundance of *Mortierella* and unclassified Basidiomycota was higher in CRYE compared to the REF while the relative abundance of unclassified Hypocreales, *Fusarium*, *Calvatia*, *Acremonium*, and *Lectera* was highest in REF compared to CRYE. Unclassified Basidiomycota, *Cystofilobasidium*, *Cryptococcus*, and *Lectera* were more abundant in PCRO than in REF while *Mortierella*, unclassified Hypocreales, *Fusarium*, *Calvatia*, and *Acremonium* were more abundant in REF compared to PCRO.

**Figure 4:**
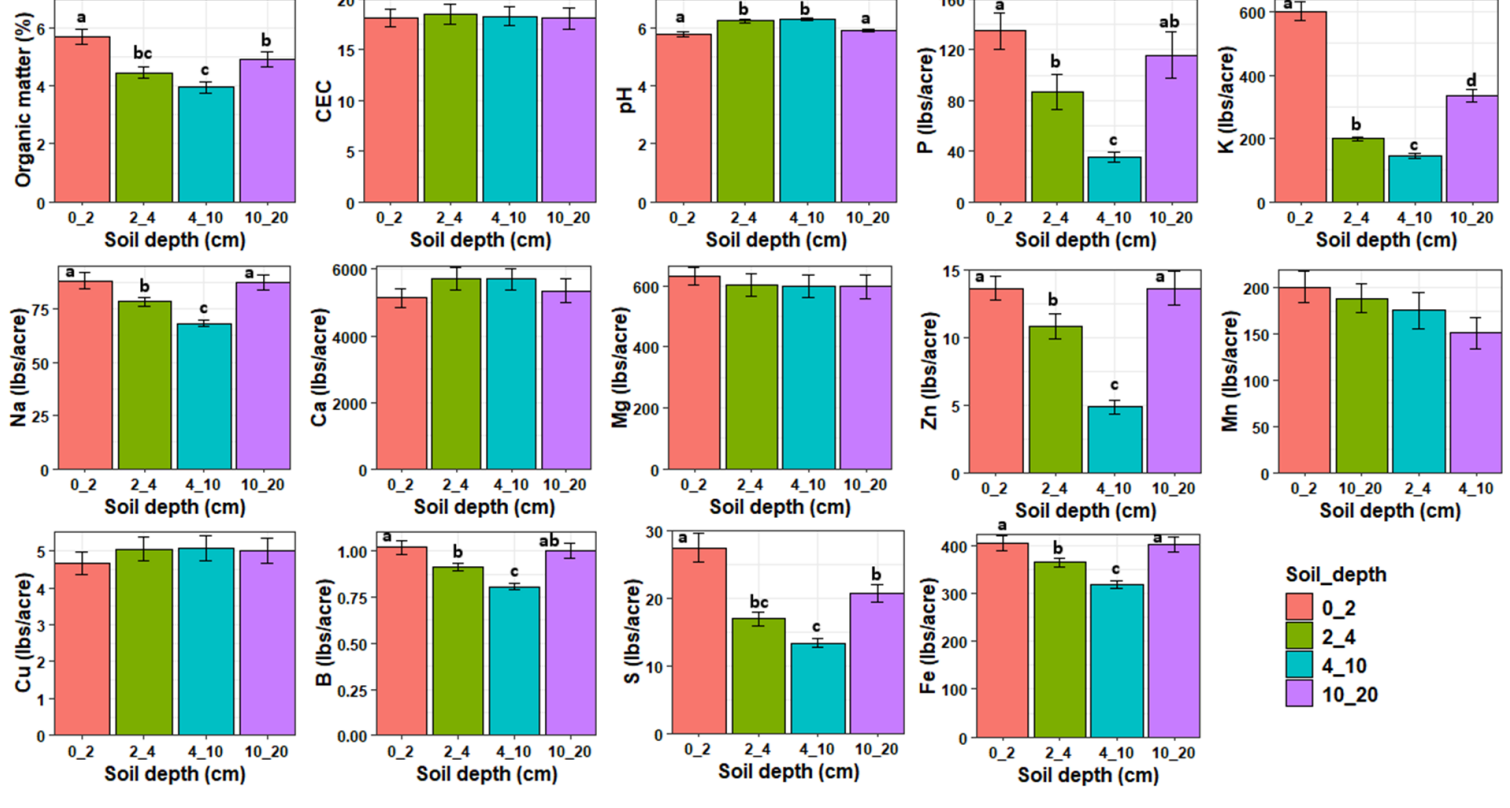
Means (±SE) of the soil physical and chemical properties in relation to soil depth across all treatments. Different letters indicate significant differences and bars without letters are not statistically significant. Values are either in pounds/acre (lbs/acre) or in percentage per kilogram of soil except for CEC whose values are in meq/100 g soil.

MANOVA revealed that the soil properties at the experimental plots were significantly influenced by soil depth (Pillai = 1.68, df = 3, 48, *p* < 0.0001) and treatment (Pillai = 1.23, df = 3, 48, *p* < 0.0001) but not their interaction (Pillai = 1.72, df = 9, 48, *p* = 0.99, Figure 4). Organic matter, P, K, Na, Zn, B, S, and Fe decreased with increasing soil depth from 0 to 10 cm depth and then increased from 10–20 cm to levels lower than or similar to those observed at 0–2 cm depth. Soil pH was significantly higher at 2–4 and 4–10 cm depths compared to 0–2 and 10–20 cm depths. There was no significant effect of soil depth on CEC, Ca, Mg, Mn, and Cu. After removing highly correlated variables, an NMDS analysis revealed that OM (r^2^ = 0.71, *p* < 0.01), K (r^2^ = 0.40, *p* < 0.01), S (r^2^ = 0.71, *p* < 0.01), P (r^2^ = 0.27, *p* < 0.01), Zn (r^2^ = 0.24, *p* < 0.01), pH (r^2^ = 0.10, *p* < 0.01), and B (r^2^ = 0.10, *p* < 0.05) were significantly associated with fungal community composition (Figure 3). pH was positively correlated with composition of deeper soil samples while OM, K, S, P, and Zn were positively correlated with the composition of topsoil samples. Phosphorus was the only variable that was significantly affected by cover crop treatment with PCRO having significantly higher phosphorus content compared to WPEN (Figure S1).

### 3.4. Prediction of Community Functions

**Figure 5:**
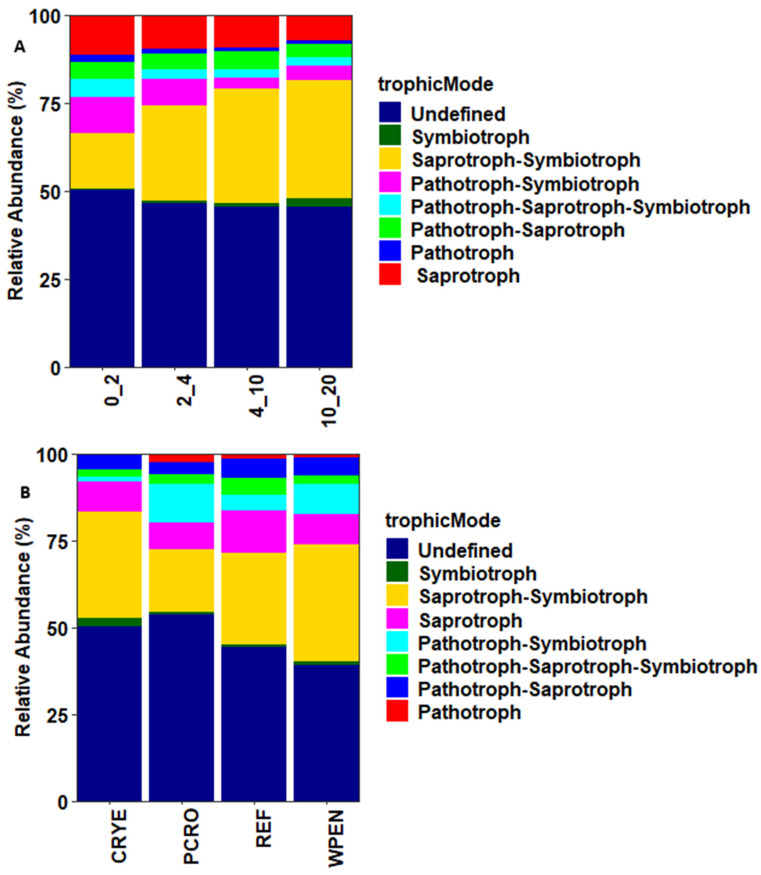
Trophic modes of fungal communities across (**A**) four soil depths and (**B**) four cover crop treatments. CRYE (cereal rye), PCRO (a mixture of pea, crimson clover, radish, and oat), REF (winter fallow reference), and WPEN (wild pennycress).

FUNGuild was used to assign soil fungal communities to Pathotrophs, Saprotrophs, Symbiotrophs, and multi-functional fungi based on their functional guilds (Figure 5). Saprotrophs were the most abundant guild, accounting for 9.4% (ranging from 7.2 to 11.5%) of the total sequences and their relative abundance decreased with increasing soil depth. Pathotrophs were the second-most abundant guild, accounting for 1.4% (ranging from 1.0 to 2.1%) of the total sequences, and was more abundant at 0–2 cm depth. Communities assigned to Symbiotrophs accounted for 1.2% (ranging from 0.3 to 2.6%) of the total sequences and their relative abundance increased with increasing soil depth. Most sequences were assigned to multiple guilds (Figure 5). These included Saprotroph–Symbiotroph (27.2%), Pathotroph–Symbiotroph (6.3%), Pathotroph–Saprotroph (4.6%), and Pathotroph–Saprotroph–Symbiotroph (3.0%). The relative abundance of Saprotroph–Symbiotroph guilds increased with increasing soil depth while that of Pathotroph–Symbiotroph decreased with increasing soil depth. Arbuscular mycorrhizal (AM) were the most dominant Symbiotrophs and their relative abundance increased with increasing soil depth: 0.2%, 0.5%, 1.3%, and 2.5% at 0–2, 2–4, 4–10, and 10–20 cm depth, respectively.

## 4. Discussion

Our data show that soil depth altered fungal community composition but had little effect on fungal diversity and richness which is inconsistent with our first hypothesis. Beta dispersion differed among groups, so the results may partly reflect differences in multivariate spread. Fungal diversity and richness commonly decrease with increasing soil depth [12,13,14,41,42,43], but several studies have reported the lack of significant differences across soil depths [44,45]. Differences in fungal community composition between deeper soil layer (10–20 cm) and the topmost layer (0–4 cm) is also commonly reported in soil microbiome studies [13,14,42]. These differences are attributed to changes in the physical and chemical properties of the soil with depth, and a decrease in available resources leading to vertical niche partitioning among fungal taxa [46,47]. Our results are consistent with these findings as we identified soil OM, K, S, P, Zn, B, and pH to be significantly associated with soil depth. The topmost layer was characterized by low pH and high contents of OM, K, S, P, Zn, and B compared to deeper soil layer. Vertical stratification of these nutrients/minerals may contribute to differences in fungal community composition as different fungal taxa differ in their niche preferences and nutrient acquisition strategies [48].

The three most abundant fungal phyla were Ascomycota (36.1%), Mortierellomycota (20.5%), and Basidiomycota (20.4%) which is consistent with previous results from the same field plots [15] and other soil ecosystems [45]. The observed decrease in the relative abundance of Ascomycota and an increase in the relative abundance of Mortierellomycota with increasing soil depth could be attributed to differences in nutrient acquisition strategies between members of the two phyla. Most members of Ascomycota are saprotrophic and produce a wide range of enzymes that degrade recalcitrant substrates such as cellulose, keratin, and lignin [49]. This enables them to thrive in the upper soil layer which is typically supplied with large quantities of crop residue by aboveground vegetations. Thus, the high abundance of several members of Ascomycota such as *Fusarium*, *Idriella*, *Plectoctophaerella*, and *Lectera* in the upper soil layers is not surprising. The genus *Fusarium* is known to contain some beneficial species as well as devastating plant pathogens and tends to grow and survive better in crop residues [42]. As the quality and quantity of leaf litter decreases at lower soil depths, other fungi including symbionts and Saprotrophs, with the ability to degrade older recalcitrant carbon sources (e.g., lignin) such as *Mortierella* and *Solicoccozyma,* may become dominant [13].

Most fungal taxa (41.2%) were assigned to more than one guild but the few taxa that were classified as strict saprophytes (9.4%) or symbionts (1.2%) responded as predicted in our second hypothesis. Saprophytes decreased with increasing soil depth while symbionts increased with increasing soil depth. Symbiotic fungi are more abundant in deeper soil layers and obtain nutrients from root secretions [50]. Arbuscular mycorrhizal (AM), the most abundant symbionts identified in this study, have previously been shown to be more abundant in the 5–10 cm and >10 cm soil depths compared to 0–5 cm depth [51], but there are also studies documenting a decrease in the relative abundance of AM with increasing soil depth [47,52]. The relative abundance of AM has been shown to decrease as the available nutrients increase [53,54]. Our results are consistent with these findings as the deeper soil layer had lower contents of K, S, P, Zn, and B than the top layer. The high abundance of Pathotrophs in the 0–2 cm depth compared to other soil depths contradicts our second hypothesis. However, most of the identified Pathotrophs were plant pathogens and high abundance of plant fungal pathogens in the upper soil layer is commonly reported [15,42,55]. These results should be interpreted with caution as the conclusions are based on a relatively small subset of the dataset (12%) consisting of fungal taxa that were only assigned to a single guild.

Fungal community composition in CRYE and PCRO treatment were significantly different from those of the REF but cover crop treatment had limited effect on soil fungal diversity and richness. Our previous studies in the same field plots found that all three cover crops enhanced soil fungal community richness relative to the REF and one cover crop (CRYE) altered fungal community composition [15]. Other studies have demonstrated that cover crops alter soil fungal community composition, but their effect on fungal diversity and richness is not consistent. Some studies reported that cover crops altered fungal community composition without affecting fungal diversity [6,29] while others reported that cover crops altered soil fungal community composition and enhanced fungal diversity [9]. Still, other studies found that cover crops enhanced soil fungal richness, reduced fungal diversity, and altered fungal community composition [10]. Further studies are needed to identify the mechanisms responsible for the variable effects of cover crops on fungal diversity and richness and to understand how cover crop-induced changes in soil fungal community composition affect crop yields and the prevalence of economically important plant pathogens in different cropping systems.

The most consistent difference between CRYE vs. REF and PCRO vs. REF was the high abundance of *Fusarium* in REF compared to either CRYE or PCRO. Other striking differences included the high abundance of Saprotrophs and Symbiotrophs such as *Mortierella*, *Filobasidium*, and Glomeraceae in CRYE relative to REF and the high abundance of potential plant pathogen *Lectera* in REF relative to CRYE. *Mortierella* is known to promote plant health by increasing availability of nitrogen and phosphorus in the rhizosphere, and by stimulating the production of phytohormones and phytoalexins [56,57]. Previous studies have reported the potential for species in this genus to be harnessed as biocontrol agents for *Fusarium* pathogens [57], but it is unclear whether the high abundance of this genus in CRYE contributed to the low abundance of *Fusarium* in this cover crop treatment. We are also aware that *Fusarium* is a diverse genus consisting of species with pathogenic, saprotrophic, and symbiotic lifestyles, and further studies are needed to characterize this genus at the species level to ascertain their trophic guild.

The findings of this study did not support our third hypothesis that different cover crop species would have variable effects on fungal communities at different soil depths. Plants influence soil fungal community assembly by providing plant litter and root exudates that serve as nutrient sources for fungi. The quality, quantity, and chemical composition of plant litter and root exudates differ among plant species which may have variable effects on different fungal taxa [58,59,60]. Plant species also differ in their rooting system, ranging from shallow to deep-reaching systems. Shallow-rooted plant species may be restricted in their physical ability to deposit microbial substrates and nutrients in deeper soil layers [61]. Thus, different plant species would be expected to have variable effects on soil fungal communities at different soil layers. While these findings are commonly reported in other systems, the interactive effect of soil depth and cover crop species on soil fungal communities is rarely investigated. A study by Zhang and colleagues reported a significant interaction between cover crop species and soil depth on soil fungal communities [62], while another study in agricultural grassland failed to detect a significant interaction between plant community composition and soil depth on soil fungal communities [61].

## 5. Conclusions

Cover cropping is attracting interest as a vital component of sustainable management of pests and diseases. Our study revealed that soil depth significantly shapes fungal community composition, likely driven by vertical gradients in soil properties such as organic matter, K, S, P, Zn, B, and pH. CRYE and PCRO cover crops were also found to modulate fungal community composition relative to the REF by increasing the abundance of beneficial genera such as *Mortierella* and suppressing other genera like *Fusarium*, which contains some devastating plant pathogens. The absence of significant interaction between soil depth and cover crop treatment suggests that each factor acts as a separate driver of soil fungal communities. However, the results should be interpreted with caution as the study had several limitations. The data was collected at a single study site on a single sampling date, which may limit the spatial and temporal generalization of the results. Soil sampling was also conducted on a limited soil depth range and, due to experimental design constraints, the “block” factor was not included in some analyses, likely affecting the robustness of those specific findings. Finally, the ITS sequence data was not calibrated to actual fungal biomass and thus, absolute quantities of fungal communities were not captured. Despite these drawbacks, the findings underscore the importance of soil depth and agricultural practices in structuring soil fungal communities in agricultural soils and could inform the development of new strategies that exploit cover crops and soil functions to enhance soil sustainability.

## Figures and Tables

**Table 1:** Alpha diversity estimates at different soil depths. Different lower-case letters indicate significant differences between means.

Soil Depth (cm)	Observed ASVs	Shannon Diversity Index
0–2	306.63 ± 17.73 ^a^	3.87 ± 0.13 ^a^
2–4	321.81 ± 9.34 ^a^	4.07 ± 0.11 ^ab^
4–10	308.44 ± 10.67 ^a^	4.36 ± 0.07 ^b^
10–20	308.44 ± 8.50 ^a^	4.20 ± 0.10 ^ab^

**Table 2:** SIMPER analysis identifying the contribution of each fungal genera to the Bray–Curtis metric across soil depths and cover crop treatments. CRYE (cereal rye), PCRO (a mixture of pea, crimson clover, radish, and oat), REF (winter fallow reference), and WPEN (wild pennycress).

				Mean Abundance			
Taxon	Av. Dissim	Contrib. %	Cumulative %	0–2 cm	2–4 cm	4–10 cm	10–20 cm
**Soil depth**							
*Mortierella*	9.2	19.2	19.2	8.8	19.8	22.7	24.9
Fungi_sp	4.4	9.2	28.4	17.2	12.3	12.5	14.7
Hypocreales	2.7	5.6	34.0	2.3	5.9	4.0	1.5
*Cystofilobasidium*	2.2	4.5	38.5	2.4	2.7	1.6	0.9
*Cryptococcus*	1.8	3.8	42.3	3.2	1.3	1.1	0.5
Basidiomycota	1.8	3.8	46.1	0.1	0.4	2.8	3.3
*Fusarium*	1.7	3.6	49.6	4.8	2.6	2.1	1.9
*Calvatia*	1.6	3.4	53.0	3.8	0.8	0.1	0.1
*Idriella*	1.5	3.1	56.1	3.3	0.7	0.3	0.3
*Metarhizium*	1.3	2.7	58.8	0.3	2.8	1.2	0.7
*Acremonium*	1.2	2.4	61.2	2.3	1.9	2.5	1.2
*Lectera*	1.0	2.1	63.3	1.7	1.1	0.5	0.8
*Clonostachys*	0.9	1.9	65.1	0.9	1.5	1.8	1.1
*Filobasidium*	0.9	1.9	67.0	1.4	1.3	0.5	0.4
Helotiales	0.9	1.8	68.8	0.5	0.5	1.6	1.5
Glomeraceae	0.8	1.8	70.6	0.1	0.4	0.9	1.8
**Cover crops**							
**Taxon**	**Av. dissim**	**Contrib. %**	**Cumulative %**	**REF**	**CRYE**	**PCRO**	**WPEN**
*Mortierella*	8.8	18.3	18.3	17.6	24.2	12.2	22.1
Fungi_sp	4.4	9.1	27.5	14.6	14.9	15.2	12.0
Hypocreales	2.7	5.6	33.1	6.8	1.4	2.1	3.2
*Cystofilobasidium*	2.5	5.3	38.3	0.0	0.3	6.8	0.5
*Cryptococcus*	2.0	4.2	42.6	0.0	0.1	5.6	0.3
*Fusarium*	1.7	3.6	46.1	4.4	2.0	2.6	2.3
Basidiomycota	1.7	3.5	49.6	1.5	2.6	1.3	1.3
*Calvatia*	1.7	3.5	53.1	4.9	0.0	0.0	0.0
*Idriella*	1.5	3.1	56.2	0.1	0.0	0.6	3.8
*Metarhizium*	1.3	2.7	58.9	0.6	0.6	0.3	3.6
*Acremonium*	1.2	2.5	61.5	3.8	1.5	1.4	1.2
*Lectera*	1.0	2.2	63.6	1.1	0.0	2.2	0.8

## Data Availability

The data presented in this study are openly available in NCBI at https://www.ncbi.nlm.nih.gov/bioproject/PRJNA935737/, reference number PRJNA1293797.

## Supplementary Materials

The following supporting information can be downloaded at: https://www.mdpi.com/article/10.3390/jof12020100/s1, Figure S1: Means (± SE) of the soil physical and chemical properties in relation to cover crop treatment. Values are either in pounds/acre (lbs/acre) or in percentage per kilogram of soil except for CEC whose values are in meq/100 g soil.
