# Supplementary material for: Fungal Community Composition and Diversity Across Soil Depths Under Different Cover Crop Treatments
Source: J Fungi (Basel). 2026 Jan 31;12(2):100. doi: 10.3390/jof12020100 (PMC12941469; doi:10.3390/jof12020100)

Supplementary Materials

Figure S1. Means (± SE) of the soil physical and chemical properties in relation to cover crop treatment. Values are either in pounds/acre (lbs/acre) or in percentage per kilogram of soil except for CEC whose values are in meq/100 g soil.

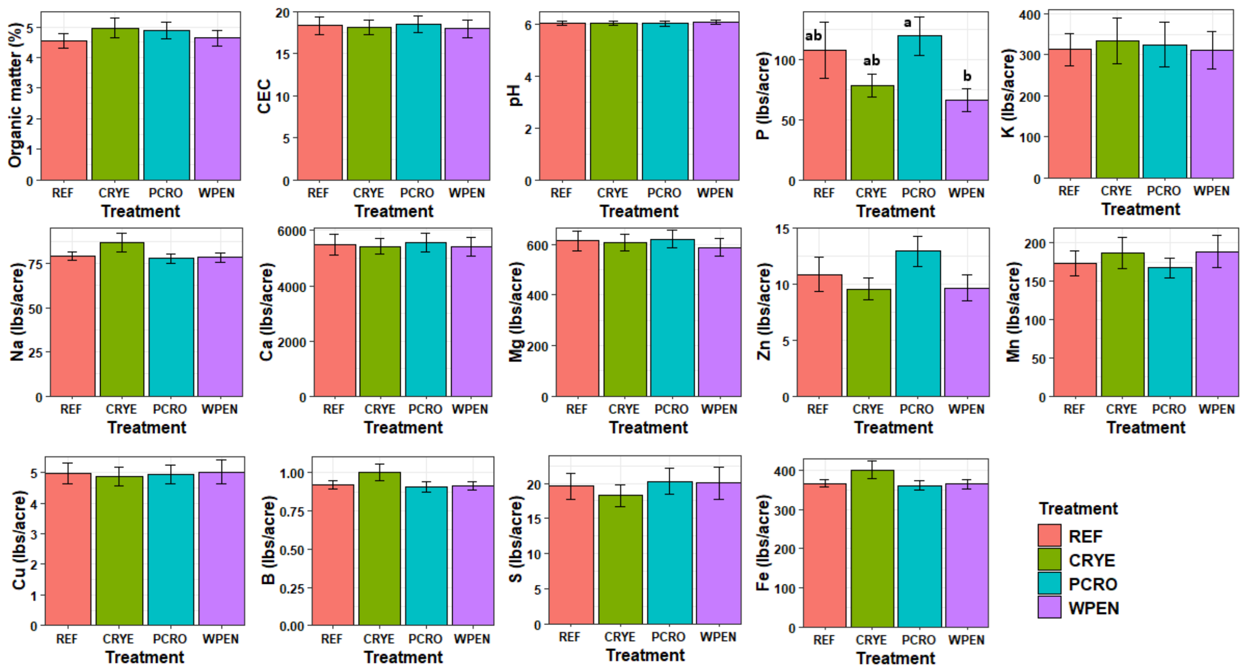

Supplement: Supplementary file 1 [file jof-12-00100-s001.zip › jof-4026371-supplementary.pdf]
